# Glaucoma in mucopolysaccharidoses

**DOI:** 10.1186/s13023-021-01935-w

**Published:** 2021-07-15

**Authors:** Weijing Kong, Jing Zhang, Cheng Lu, Yingxue Ding, Yan Meng

**Affiliations:** 1 Department of Pediatrics, Beijing Friendship Hospital, Capital Medical University, Beijing, 100050 China; 2 Department of Pediatrics, Chinese PLA General Hospital, Beijing, 100853 China; 3 Beijing Hong Jian Medical Device Company, Beijing, 100176 China

**Keywords:** Mucopolysaccharidoses, Mucopolysaccharidosis, Glaucoma, Glycosaminoglycans, Rare disease, Lysosomal storage disorders

## Abstract

Mucopolysaccharidoses are a group of lysosomal storage disorders that are caused by deficiency of enzymes involved in glycosaminoglycans degradation. Due to low prevalence and high childhood mortality, researches on mucopolysaccharidoses were mainly focused on the fatal manifestations. With the development of treatments, more and more mucopolysaccharidoses patients were treated by approved therapies, thereby getting prolonged life span and improved quality of life. Abnormal accumulation of glycosaminoglycans in the eye may block trabecular meshwork, thicken sclera and change mechanical behavior of lamina cribrosa, which, by increasing intraocular pressure and damaging optic nerve, could cause glaucoma. Glaucoma was the leading cause of irreversible blindness worldwide, but it was rarely reported in mucopolysaccharidoses patients. Although non-fatal, it seriously affected quality of life. Prevalence of glaucoma in mucopolysaccharidoses patients (ranged from 2.1 to 12.5%) indicated that glaucoma in patients with mucopolysaccharidoses was worthy of attention and further study, thereby improving the quality of life for MPSs patients.

## Background

Mucopolysaccharidoses (MPSs) are rare lysosomal storage disorders that are caused by abnormal accumulation of glycosaminoglycans (GAGs), which is due to deficiency of enzymes involved in degradation of GAGs [1]. MPSs are classified into seven subtypes. Six subtypes of MPSs (type I, III, IV, VI, VII and IX) are inherited in an autosomal recessive manner, while mucopolysaccharidosis (MPS) II is X-linked [2]. Milder forms of MPS I and II, MPS IV, and MPS VI are not considered to be progressive or neuronopathic, although patients may function abnormally in neurocognitive ability and/or behavior [3].

Due to low prevalence and high childhood mortality of MPSs, researches were mainly focused on the fatal manifestations. Governments typically stimulated development of specific therapies for MPSs by providing regulatory and economic incentives. For example, enzyme replacement therapies for non-neuronopathic forms of MPS I and II have been developed and approved to date [4]. Life span and quality of life were improved after the treatment [5, 6]. Non-fatal manifestations of patients with MPSs, i.e. ocular manifestations, should be noticed and intervened earlier to get better prognosis. Ophthalmological findings (corneal clouding, glaucoma, optic neuropathies, and retinopathies) were common and variable in MPSs and might result in significant visual impairment [1, 7].

Glaucoma, as a leading cause of irreversible blindness, is a group of eye conditions that are characterized by progressive degeneration of retinal ganglion cells [8, 9]. Glaucoma was untreatable, but the rate of visual field deterioration could be slowed down by reducing intraocular pressure (IOP) [10]. Prevalence of glaucoma in MPSs patients (ranged from 2.1% to 12.5%) suggested that glaucoma in patients with MPSs was worthy of attention [11]. This review was published to attract more attention from people about glaucoma in patients with MPSs and aimed to increase efforts in improving the quality of life for MPSs patients.

### Glaucoma in MPSs

Glaucoma was known to be related with MPS I [12–17], MPS IV [18] and MPS VI [19–22], but it was rarely reported in the other subtypes of MPS. Until 2015, there was only one MPS II patient that was diagnosed with suspected glaucoma [23]. Ashworth et al. (2015) reported the first case series to assess and diagnose suspected glaucoma in patients with MPSs and to determine its prevalence [11]. In the report of Ashworth et al. (2015), there were 4 patients with MPS I, 2 patients with MPS II, 1 patient with MPS IVA, and 7 patients with MPS VI [11]. To the best of our knowledge, only one case with MPS III was reported to have glaucoma [24], while patients with MPS VII and IX were never reported to show glaucoma.

### Possible pathogenesis of glaucoma in MPSs

Clinical manifestations of MPSs were caused by abnormal accumulation of GAGs, which were long and unbranched heteropolymers with repeating disaccharide units that were made up of carbohydrate moiety of proteoglycans [25]. GAGs were widespread both in extracellular matrix and at cell surface. Biological functions of GAGs included regulation of cell growth and differentiation, tissue hydration maintenance and structure stabilization [26]. Distribution and function of GAGs in the development, homeostasis and pathology of ocular surface were discussed by Puri et al. (2020), clarifying how GAGs correlated with pathology [26]. Maric et al. (2019) revealed that newly diagnosed glaucoma patients had higher concentration of GAGs than those without glaucoma and implicated the relationship between GAGs and glaucoma [27].

**Fig. 1:**
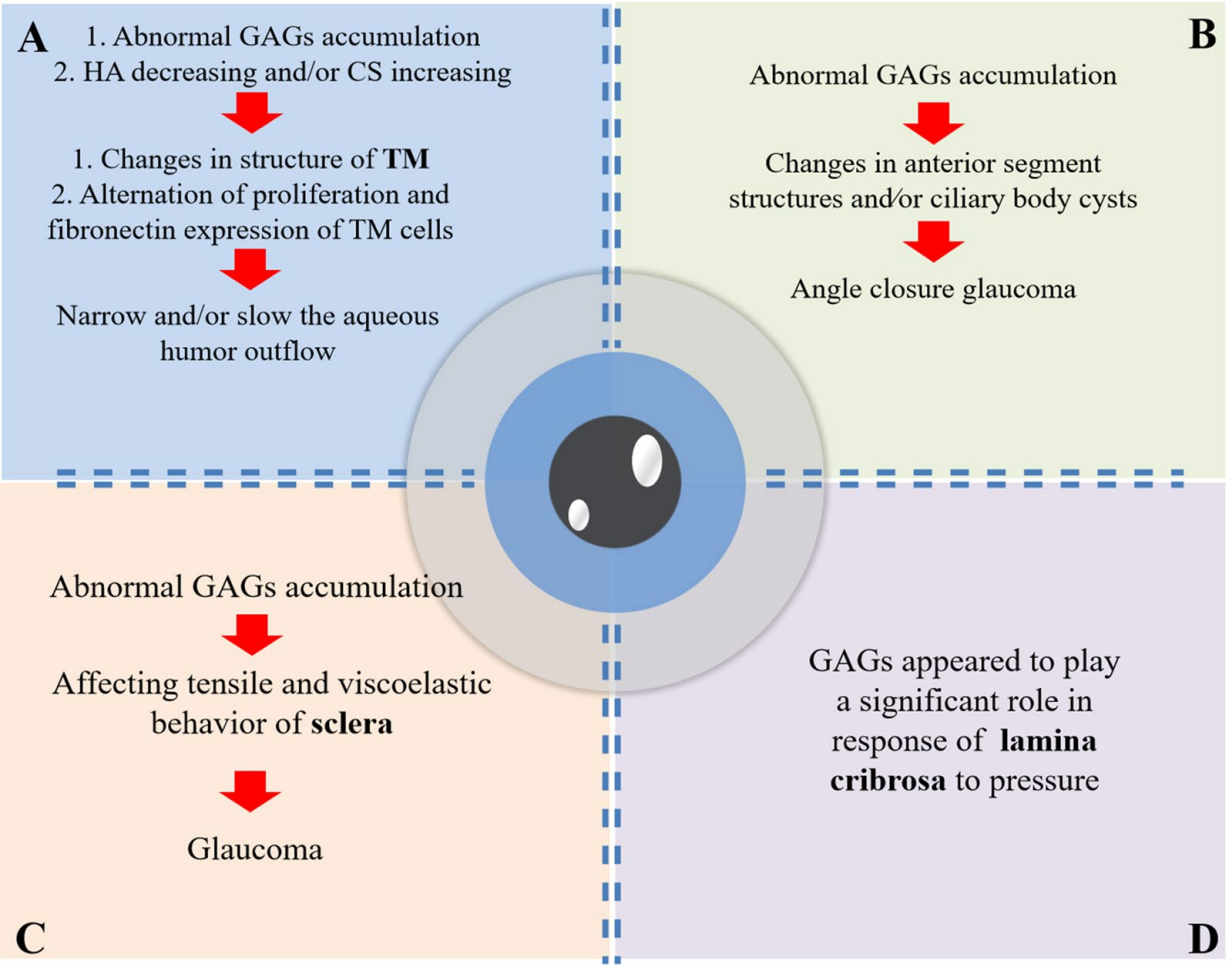
Possible pathogenesis of glaucoma in mucopolysaccharidoses patients. *GAGs* glycosaminoglycans, *HA* hyaluronic acid, *CS* chondroitin sulfate, *TM* trabecular meshwork

The main risk factor for glaucoma is increased IOP, which may be caused by dysfunction of trabecular meshwork (TM) (Fig. 1A). TM locates within iridocorneal angle and is the main pathway for drainage of aqueous humor [28]. In 1954, Barany found that perfusion of aqueous outflow system with testicular hyaluronidase (degrading enzyme for GAGs) could increase facility of outflow [29]. The interesting phenomenon attracted lots of efforts to explore morphological features and biochemical values of GAGs in the pathway for drainage of aqueous humor.

Numerous GAGs and proteoglycans were expressed in TM [30]. At least six distinct classes of GAGs were recognized in TM: chondroitin sulfate (CS), dermatan sulfate (DS), heparan sulfate (HS), heparin sulfate (Hep), keratan sulfate (KS), and hyaluronic acid (HA, also known as hyaluronan) [31]. Five of them were related with MPSs [6]. Different kinds of GAGs performed different functions in TM. HA decrease and/or CS increase could narrow and/or slow the aqueous humor outflow [32]. The phenomenon was also observed in other reports about glaucomatous eyes of rabbits and humans [33, 34].

The relationship between GAGs and TM was not only studied at the animal level, but also explored at the cell level. Proliferation and fibronectin expression of TM cells were affected by pore size, alignment and composition of GAGs. This work provided insight into how the architecture and composition of collagen-GAGs scaffolds affected TM cells behavior [35].

Open angle glaucoma may be caused by abnormal GAGs deposition within TM, while angle closure glaucoma may result from abnormal accumulation of GAGs in anterior segment structures and/or ciliary body cysts [36, 37] (Fig. 1B). Glaucoma was firstly reported by Quigley et al. (1975) in two siblings with MPS I-S, who showed a sudden increase in IOP [16]. Quigley et al. (1975) speculated that the thickening of anterior ocular structures due to abnormal storage of acid mucopolysaccharide was the reason for angle closure glaucoma [16]. In patients with MPS VI, ultrasound images revealed that angle closure glaucoma was induced by shallow anterior chamber and thickened cornea with very thick retinal-choroidal-scleral [22].

Coudrillier et al. (2012) reported that thickness and biomechanical response differed between human glaucoma eyes and normal eyes [38]. Contribution of GAGs to tensile response of posterior sclera was confirmed in porcine and human eyes [39, 40]. GAGs may affect tensile and viscoelastic behavior of sclera and then causes glaucoma; however, more evidence in support of this speculation is needed (Fig. 1C).

Evaluation of optic nerve head (ONH), where retinal ganglion cell axons exit in the eye, is important for diagnosis and management of glaucoma [41]. Stress and strain state of ONH is strongly influenced by IOP and mechanical properties of lamina cribrosa [42]. Lamina cribrosa, the weakest part of sclera, bulges outward when IOP is raised chronically in the condition of glaucoma [43]. Contribution of GAGs to mechanical behavior of human lamina cribrosa was investigated by ex vivo inflation test. Results showed that GAGs were critical for the response of lamina cribrosa to pressure [44]. Tezel et al. (1999) observed increased levels of autoantibodies recognizing GAGs in lamina cribrosa of glaucomatous eyes; however, the precise role played by autoantibodies for GAGs in ONH of glaucoma patients requires more research [45] (Fig. 1D).

### Diagnosis of glaucoma in MPSs

Patients with MPSs could develop glaucoma due to abnormal accumulation of GAGs, yet it was rarely reported in those patients. The following reasons could account for this phenomenon: (1) life span of patients with MPSs was usually not long enough to show obvious clinical manifestations of glaucoma; (2) non-ocular clinical manifestations of MPSs, including cognitive impairment and bone diseases, hindered diagnosis of glaucoma; (3) corneal clouding could block examination of inner contents of eyes and affect IOP measurement.

Age is an established risk factor for glaucoma [46]. Except for a few mild cases, MPSs is ultimately fatal with an average life expectancy of one to two decades if untreated [47]. MPSs patients could not live long enough to show obvious clinical manifestations of glaucoma, which may cause doctors to ignore this non-fatal manifestation. Limited language of young age patients with MPSs made it impossible for them to communicate changes in vision. More and more MPSs patients with glaucoma would be diagnosed, because the development of treatment would contribute to improvement of life span.

Non-ocular clinical manifestations of MPSs were also obstacles to diagnosis of glaucoma. Clinical manifestations of MPSs that blocked communication, including hearing loss and mental-retardation, may hinder doctors from understanding the condition of patients with MPSs [2, 48]. MPSs patients with bone diseases may have difficulty in posing for evaluation/imaging [49].

**Fig. 2:**
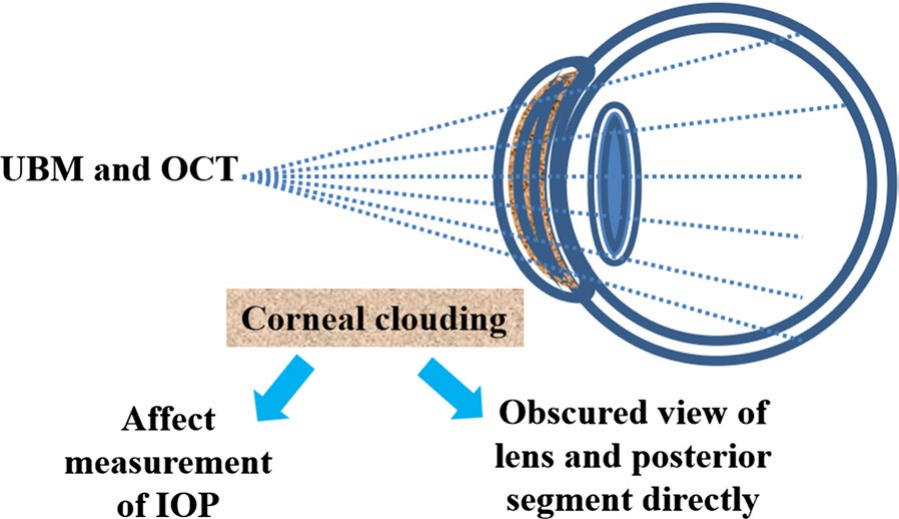
Corneal clouding blocked diagnosis of glaucoma. *UBM* ultrasound biomicroscopy, *OCT* optical coherence tomography, *IOP* increased intraocular pressure

Corneal clouding, which was caused by abnormal accumulation of GAGs in corneal, did not block communication but could hinder diagnosis of glaucoma [50]. It was common in all subtypes of MPS but more frequent in MPS IH, MPS IH-S, MPS VI, and MPS VII [50]. Corneal clouding was found in approximately 70% of patients, with median age of 4 years old for MPS I H/S and 10 years old for MPS IS [51]. Lin et al. (2019) reported that all patients with MPS I and MPS VI and 94% of MPS IV patients had various degrees of corneal opacity in their retrospective research [52]. Corneal clouding did not only block the examination of the lens and posterior segment (vitreous and retina), but also affected IOP measurement by changing corneal thickness (Fig. 2). Wasielica-Poslednik et al. (2015 and 2017) confirmed positive correlation between corneal opacity and value of IOP. Meanwhile, they reported that IOP-values of eyes with strongly affected corneas (grade 4 in MPSs) were overestimated [53, 54]. Based on the conclusion, IOP-values in reports of Lin et al. (2019) may be overestimated [52].

IOP should be tested by the same, individually adjusted and well-tolerated devices during follow-up of MPSs patients since different tonometry methods may result in variable IOP-values. Goldmann applanation tonometry, the gold standard for IOP measurement, was less dependent on corneal properties, making it more reliable for measuring IOP of patients with MPSs [55]. Ocular response analyzer and iCare rebound tonometer were tested and proved to be attractive alternatives to applanation tonometry in MPS patients [54]. Ultrasound biomicroscopy and optical coherence tomography played an increasingly important role in diagnosis of glaucoma in MPSs patients, because they could provide detailed views of anatomy behind cornea clouding (Fig. 2) [56].

A group of paediatric ophthalmologists, an orthoptist and an optometrist with extensive experience in children with MPSs, held a meeting in Stockholm in September 2010 and drew up clinical guidelines for diagnosis and management of ocular manifestations of MPSs [57]. The guidelines gave an overview of basic ocular assessments and a number of optional tests for children with MPSs. Diagnosis of glaucoma in MPSs patients could also follow European glaucoma society terminology and guidelines for glaucoma (4th Edition) [58, 59].

### Management of glaucoma of MPSs

Treatments for glaucoma in MPSs patients could follow the clinical guidelines for glaucoma: (1) initial therapy was topical medication or laser trabeculoplasty; (2) if patients failed to attain the target IOP during follow-up, additional therapies should be considered, such as trabeculectomy, non-penetrating deep sclerectomy and/or other glaucoma surgeries [60]. In addition to traditional treatments for glaucoma, hematopoietic stem cell transplantation (HSCT) for MPS-IH patients may show good results for glaucoma treatment, though it needs more exploration [61]. Although enzyme replacement therapy (ERT) was safer than HSCT, effect of ERT on ocular manifestations was limited and variable [62]. Corneal clouding of MPS I and MPS VI patients could remain stable after ERT [63–65]. ERT also could maintain stability of sclera thickness of MPS I and MPS VI patients [66]. ERT was also tried to treat glaucoma in MPS VI patients; however, no changes were observed [67]. Retina–brain barrier and the avascular nature of cornea may reduce clinical efficacy of ERT for treating eye pathology. With the development of treatment, prevalence of glaucoma would be higher, because MPSs patients who were treated by approved therapies would get longer life span.

Current knowledge about the benefits and risks of anti-glaucoma therapies for MPSs patients is limited, because MPSs patients with glaucoma were rarely reported. A multicenter retrospective case note review reported the impact of medical treatments on IOP of 12 eyes from MPSs patients with glaucoma: IOP of 7 eyes was reduced; 1 eye was less successfully treated; 2 eyes stopped receiving treatment; IOP of 1 eye was reduced after keratoplasty [11]. The results were compliant with former reports: some reports showed improvements in IOP or vision after anti-glaucoma treatments [14, 15]; however, other reports showed that anti-glaucoma treatments were not good enough [17].

## Limitation

(1) Reports of glaucoma in MPSs patients were rare; reports about results of anti-glaucoma therapies for MPSs patients with glaucoma were rarer. Limited data hampered efforts to have a full view of prevalence of glaucoma in MPSs patients, benefits and risks of anti-glaucoma therapies, among other aspects. (2) Pathogenesis of glaucoma in MPSs patients was not clear, although a handful of reports explored the pathogenesis.

## Conclusion

Abnormal accumulation of GAGs may cause glaucoma in MPSs patients by affecting functions and structures of eye, including TM, cornea, ciliary body and sclera. Clinical manifestations and shortened life span could hamper diagnosis of glaucoma in MPSs patients and block knowledge accumulation on the benefits and risks of anti-glaucoma therapies. Despite the fact that cases of glaucoma in MPSs patients were rarely reported, prevalence of glaucoma in MPSs patients (ranged from 2.1% to 12.5%) indicated that glaucoma in MPSs patients was worthy of attention and further study so that quality of life for MPSs patients could be improved.

## Data Availability

Data can be made available from the corresponding author after discussion with the Institutional Review Board.

## Abbreviations

MPSs: Mucopolysaccharidoses
GAGs: Glycosaminoglycans
MPS: Mucopolysaccharidosis
IOP: Intraocular pressure
TM: Trabecular meshwork
CS: Chondroitin sulfate
DS: Dermatan sulfate
HS: Heparan sulfate
Hep: Heparin sulfate
KS: Keratan sulfate
HA: Hyaluronic acid
ONH: Optic nerve head
HSCT: Hematopoietic stem cell transplantation
ERT: Enzyme replacement therapy

## References

[1] Tomatsu S, Pitz S, Hampel U (2019). Ophthalmological Findings in Mucopolysaccharidoses. J Clin Med.

[2] Wolfberg J, Chintalapati K, Tomatsu S, Nagao K (2020). hearing loss in mucopolysaccharidoses: current knowledge and future directions. Diagnostics (Basel).

[3] Shapiro EG, Eisengart JB (2021). The natural history of neurocognition in MPS disorders: a review. Mol Genet Metab.

[4] Tambuyzer E, Vandendriessche B, Austin CP, Brooks PJ, Larsson K, Miller Needleman KI, Valentine J, Davies K, Groft SC, Preti R (2020). Therapies for rare diseases: therapeutic modalities, progress and challenges ahead. Nat Rev Drug Discov.

[5] Parini R, Deodato F (2020). Intravenous Enzyme Replacement Therapy in Mucopolysaccharidoses: Clinical Effectiveness and Limitations. Int J Mol Sci.

[6] Fecarotta S, Gasperini S, Parenti G (2018). New treatments for the mucopolysaccharidoses: from pathophysiology to therapy. Ital J Pediatr.

[7] Sornalingam K, Javed A, Aslam T, Sergouniotis P, Jones S, Ghosh A, Ashworth J (2019). Variability in the ocular phenotype in mucopolysaccharidosis. Br J Ophthalmol.

[8] Kwon S, Kim SH, Khang D, Lee JY (2020). Potential therapeutic usage of nanomedicine for glaucoma treatment. Int J Nanomed.

[9] Weinreb RN, Aung T, Medeiros FA (2014). The pathophysiology and treatment of glaucoma: a review. JAMA.

[10] Society EG: European Glaucoma Society Terminology and Guidelines for Glaucoma, 4th Edition - Chapter 3: Treatment principles and options Supported by the EGS Foundation: Part 1: Foreword; Introduction; Glossary; Chapter 3 Treatment principles and options. *Br J Ophthalmol* 2017, 101(6):130–195. 10.1136/bjophthalmol-2016-EGSguideline.003. 10.1136/bjophthalmol-2016-EGSguideline.003 PMC5583689 28559477

[11] Ashworth J, Flaherty M, Pitz S, Ramlee A (2015). Assessment and diagnosis of suspected glaucoma in patients with mucopolysaccharidosis. Acta Ophthalmol.

[12] Girard B, Hoang-Xuan T, D'Hermies F, Savoldelli M, Bennouna M, Poenaru L, Maroteaux P, Pouliquen Y: [Mucopolysaccharidosis type I, Hurler-Scheie phenotype with ocular involvement. Clinical and ultrastructural study]. *J Fr Ophtalmol* 1994, 17(4):286–295 8089412

[13] Hamma A, Bousalah M (2016). Glaucoma and mucopolysaccharidosis type I: Report of two children. J Fr Ophtalmol.

[14] Mullaney P, Awad AH, Millar L (1996). Glaucoma in mucopolysaccharidosis 1-H/S. J Pediatr Ophthalmol Strabismus.

[15] Nowaczyk MJ, Clarke JT, Morin JD (1988). Glaucoma as an early complication of Hurler's disease. Arch Dis Child.

[16] Quigley HA, Maumenee AE, Stark WJ (1975). Acute glaucoma in systemic mucopolysaccharidosis I-S. Am J Ophthalmol.

[17] Spellacy E, Bankes JL, Crow J, Dourmashkin R, Shah D, Watts RW (1980). Glaucoma in a case of Hurler disease. Br J Ophthalmol.

[18] Cahane M, Treister G, Abraham FA, Melamed S (1990). Glaucoma in siblings with Morquio syndrome. Br J Ophthalmol.

[19] Bergwerk KL, Rabinowitz YS, Falk RE (2003). Quality of life related to visual function in three young adults with mucopolysaccharidoses. ScientificWorldJournal.

[20] Cantor LB, Disseler JA, Wilson FM (1989). Glaucoma in the Maroteaux-Lamy syndrome. Am J Ophthalmol.

[21] Marinho D, Azevedo AC, Rymer S, Giugliani R, Schwartz IV: Pseudo-glaucoma in type VI mucopolysaccharidosis: case report. *Arq Bras Oftalmol* 2007, 70(3):563; author reply 564 doi: N/A. 17768572

[22] Veerappan M, Chak G, Shieh C, Challa P (2017). Atypical presentation of acute angle-closure glaucoma in Maroteaux-Lamy mucopolysaccharidosis with patent prophylactic laser peripheral iridotomy: a case report. Perm J.

[23] Sethi S, Bhartiya S, Mehta M, Chandra M, Ghose S. Hunter’s syndrome and buphthalmos in a girl: an unusual ophthalmic association. Acta Ophthalmol. 2010;87:244. 10.1111/j.1755-3768.2009.2148.x.

[24] Lin SC, Hu FR, Hou JW, Yao YT, Wang TR, Hung PT (1999). Corneal opacity and congenital glaucoma associated with massive heparan sulfaturia: report of one case. Acta Paediatr Taiwan.

[25] Sasarman F, Maftei C, Campeau PM, Brunel-Guitton C, Mitchell GA, Allard P (2016). Biosynthesis of glycosaminoglycans: associated disorders and biochemical tests. J Inherit Metab Dis.

[26] Wei J, Hu M, Huang K, Lin S, Du H (2020). Roles of proteoglycans and glycosaminoglycans in cancer development and progression. Int J Mol Sci.

[27] Maric VD, Bozic MM, Cirkovic AM, Stankovic SD, Marjanovic IS, Grgurevic AD (2019). Serum heparan sulfate and chondroitin sulfate concentrations in patients with newly diagnosed exfoliative glaucoma. PeerJ.

[28] Buffault J, Labbe A, Hamard P, Brignole-Baudouin F, Baudouin C (2020). The trabecular meshwork: Structure, function and clinical implications. A review of the literature. J Fr Ophtalmol.

[29] Barany EH (1954). In vitro studies of the resistance to flow through the angle of the anterior chamber. Acta Soc Med Ups.

[30] Acott TS, Kelley MJ (2008). Extracellular matrix in the trabecular meshwork. Exp Eye Res.

[31] Pescosolido N, Cavallotti C, Rusciano D, Nebbioso M (2012). Trabecular meshwork in normal and pathological eyes. Ultrastruct Pathol.

[32] Knepper PA, Goossens W, Palmberg PF (1996). Glycosaminoglycan stratification of the juxtacanalicular tissue in normal and primary open-angle glaucoma. Invest Ophthalmol Vis Sci.

[33] Knepper PA, Collins JA, Frederick R (1985). Effects of dexamethasone, progesterone, and testosterone on IOP and GAGs in the rabbit eye. Invest Ophthalmol Vis Sci.

[34] Wordinger RJ, Clark AF (1999). Effects of glucocorticoids on the trabecular meshwork: towards a better understanding of glaucoma. Prog Retin Eye Res.

[35] Osmond MJ, Krebs MD, Pantcheva MB (2020). Human trabecular meshwork cell behavior is influenced by collagen scaffold pore architecture and glycosaminoglycan composition. Biotechnol Bioeng.

[36] Sato S, Maeda N, Watanabe H, Tano Y, Inoue Y, Shimomura Y, Tanaka A (2002). Multiple iridociliary cysts in patients with mucopolysaccharidoses. Br J Ophthalmol.

[37] Ahmed TY, Turnbull AM, Attridge NF, Biswas S, Lloyd IC, Au L, Ashworth JL (2014). Anterior segment OCT imaging in mucopolysaccharidoses type I, II, and VI. Eye (Lond).

[38] Coudrillier B, Tian J, Alexander S, Myers KM, Quigley HA, Nguyen TD (2012). Biomechanics of the human posterior sclera: age- and glaucoma-related changes measured using inflation testing. Invest Ophthalmol Vis Sci.

[39] Murienne BJ, Chen ML, Quigley HA, Nguyen TD (2016). The contribution of glycosaminoglycans to the mechanical behaviour of the posterior human sclera. J R Soc Interface.

[40] Hatami-Marbini H, Pachenari M (2020). The contribution of sGAGs to stress-controlled tensile response of posterior porcine sclera. PLoS ONE.

[41] Wareham LK, Calkins DJ (2020). The neurovascular unit in glaucomatous neurodegeneration. Front Cell Dev Biol.

[42] Boote C, Sigal IA, Grytz R, Hua Y, Nguyen TD, Girard MJA (2020). Scleral structure and biomechanics. Prog Retin Eye Res.

[43] Downs JC, Girkin CA (2017). Lamina cribrosa in glaucoma. Curr Opin Ophthalmol.

[44] Midgett DE, Jefferys JL, Quigley HA, Nguyen TD (2018). The contribution of sulfated glycosaminoglycans to the inflation response of the human optic nerve head. Invest Ophthalmol Vis Sci.

[45] Tezel G, Edward DP, Wax MB (1999). Serum autoantibodies to optic nerve head glycosaminoglycans in patients with glaucoma. Arch Ophthalmology (Chicago, III: 1960).

[46] Guedes G, Tsai JC, Loewen NA (2011). Glaucoma and aging. Curr Aging Sci.

[47] Rigoldi M, Verrecchia E, Manna R, Mascia MT (2018). Clinical hints to diagnosis of attenuated forms of Mucopolysaccharidoses. Ital J Pediatr.

[48] Kong W, Meng Y, Zou L, Yang G, Wang J, Shi X (2020). Mucopolysaccharidosis III in Mainland China: natural history, clinical and molecular characteristics of 34 patients. J Pediatr Endocrinol Metab.

[49] Tomatsu S, Almeciga-Diaz CJ, Montano AM, Yabe H, Tanaka A, Dung VC, Giugliani R, Kubaski F, Mason RW, Yasuda E (2015). Therapies for the bone in mucopolysaccharidoses. Mol Genet Metab.

[50] Del Longo A, Piozzi E, Schweizer F (2018). Ocular features in mucopolysaccharidosis: diagnosis and treatment. Ital J Pediatr.

[51] Beck M, Arn P, Giugliani R, Muenzer J, Okuyama T, Taylor J, Fallet S (2014). The natural history of MPS I: global perspectives from the MPS I Registry. Genet Med.

[52] Lin HY, Chan WC, Chen LJ, Lee YC, Yeh SI, Niu DM, Chiu PC, Tsai WH, Hwu WL, Chuang CK (2019). Ophthalmologic manifestations in Taiwanese patients with mucopolysaccharidoses. Mol Genet Genomic Med.

[53] Wasielica-Poslednik J, Politino G, Schmidtmann I, Lorenz K, Bell K, Pfeiffer N, Pitz S (2017). Influence of corneal opacity on intraocular pressure assessment in patients with lysosomal storage diseases. PLoS ONE.

[54] Wasielica-Poslednik J, Butsch C, Lampe C, Elflein H, Lamparter J, Weyer V, Pitz S (2015). Comparison of rebound tonometry, perkins applanation tonometry and ocular response analyser in mucopolysaccharidosis patients. PLoS ONE.

[55] Zhang H, Sun Z, Li L, Sun R, Zhang H (2020). Comparison of intraocular pressure measured by ocular response analyzer and Goldmann applanation tonometer after corneal refractive surgery: a systematic review and meta-analysis. BMC Ophthalmol.

[56] Javed A, Aslam T, Ashworth J (2016). Use of new imaging in detecting and monitoring ocular manifestations of the mucopolysaccharidoses. Acta Ophthalmol.

[57] Fahnehjelm KT, Ashworth JL, Pitz S, Olsson M, Tornquist AL, Lindahl P, Summers CG (2012). Clinical guidelines for diagnosing and managing ocular manifestations in children with mucopolysaccharidosis. Acta Ophthalmol.

[58] Blanco AA, Bagnasco L, Bagnis A, Barton K, Baudouin C, Bengtsson B, Bron A, Cordeiro F: European Glaucoma Society Terminology and Guidelines for Glaucoma, 4th Edition - Part 1Supported by the EGS Foundation. *Br J Ophthalmol* 2017. 10.1136/bjophthalmol-2016-EGSguideline.001.

[59] Blanco AA, Bagnasco L, Bagnis A, Barton K, Baudouin C, Bengtsson B, Bron A, Cordeiro F: European Glaucoma Society Terminology and Guidelines for Glaucoma, 4th Edition - Chapter 2: Classification and terminologySupported by the EGS Foundation: Part 1: Foreword; Introduction; Glossary; Chapter 2 Classification and Terminology. *Br J Ophthalmol* 2017, 101(5):73–127. 10.1136/bjophthalmol-2016-EGSguideline.002. 10.1136/bjophthalmol-2016-EGSguideline.002 PMC5583685 28424171

[60] Blanco AA, Bagnasco L, Bagnis A, Barton K, Baudouin C, Bengtsson B, Bron A, Cordeiro F: European Glaucoma Society Terminology and Guidelines for Glaucoma, 4th Edition - Chapter 3: Treatment principles and options Supported by the EGS Foundation: Part 1: Foreword; Introduction; Glossary; Chapter 3 Treatment principles and options. *Br J Ophthalmol* 2017, 101(6):130–195. 10.1136/bjophthalmol-2016-EGSguideline.003. 10.1136/bjophthalmol-2016-EGSguideline.003 PMC5583689 28559477

[61] Aldenhoven M, Wynn RF, Orchard PJ, O'Meara A, Veys P, Fischer A, Valayannopoulos V, Neven B, Rovelli A, Prasad VK (2015). Long-term outcome of Hurler syndrome patients after hematopoietic cell transplantation: an international multicenter study. Blood.

[62] Summers CG, Fahnehjelm KT, Pitz S, Guffon N, Koseoglu ST, Harmatz P, Scarpa M (2010). Systemic therapies for mucopolysaccharidosis: Ocular changes following haematopoietic stem cell transplantation or enzyme replacement therapy - a review. Clinic Exp Ophthalmol.

[63] Pitz S, Ogun O, Bajbouj M, Arash L, Schulze-Frenking G, Beck M (2007). Ocular changes in patients with mucopolysaccharidosis I receiving enzyme replacement therapy: a 4-year experience. Arch Ophthalmol (Chicago, III: 1960).

[64] Kakkis ED, Muenzer J, Tiller GE, Waber L, Belmont J, Passage M, Izykowski B, Phillips J, Doroshow R, Walot I (2001). Enzyme-replacement therapy in mucopolysaccharidosis I. N Engl J Med.

[65] Scarpa M, Barone R, Fiumara A, Astarita L, Parenti G, Rampazzo A, Sala S, Sorge G, Parini R (2009). Mucopolysaccharidosis VI: the Italian experience. Eur J Pediatr.

[66] Schumacher RG, Brzezinska R, Schulze-Frenking G, Pitz S (2008). Sonographic ocular findings in patients with mucopolysaccharidoses I, II and VI. Pediatric Radiol.

[67] Lin HY, Huang YH, Lei SY, Chen LJ, Lin SP (2019). Clinical ocular manifestations of Taiwanese patients with mucopolysaccharidoses VI (Maroteaux-Lamy syndrome). Taiwan J Ophthalmol.

